# Neuropsychological profile in the preclinical stages of dementia: principal component analysis approach

**DOI:** 10.1590/1980-57642021dn15-020006

**Published:** 2021

**Authors:** Claudia Rivera-Fernández, Nilton Custodio, Marcio Soto-Añari

**Affiliations:** 1 Universidad Nacional de San Agustín de Arequipa ‒ Arequipa, Perú.; 2 Instituto Peruano de Neurociencias ‒ Lima, Perú.

**Keywords:** mild cognitive impairment, neuropsychology, principal component analysis, subjective cognitive decline, cognitive dysfunction, demência pré-clínica, neuropsicologia, análise de componentes principais, declínio cognitivo, comprometimento cognitivo leve

## Abstract

The preclinical stages of dementia include subtle neurocognitive changes that are not easily detected in standard clinical evaluations. Neuropsychological evaluation is important for the classification and prediction of deterioration in all the phases of dementia.

**Objective:**

Compare the neuropsychological performance in healthy older adults with subjective cognitive decline (SCD) and with mild cognitive impairment (MCI) using principal components analysis.

**Methods:**

We evaluated 94 older adults with a clinical protocol which included general measures of mental, emotional and functional state. The neuropsychological protocol included tasks of memory, executive function, attention, verbal fluency and visuoconstructional abilities. We used principal component analysis (PCA) to reduce variables´ dimensionality on neuropsychological evaluation.

**Results:**

33(35%) participants had a normal cognitive function, 35(37%) had subjective cognitive decline and 26(28%) had a mild cognitive impairment. The PCA showed seven factors: processing speed, memory, visuoconstruction, verbal fluency and executive components of cognitive flexibility, inhibitory control and working memory. ANOVA had shown significant differences between the groups in the memory (F=4.383, p=0.016, η2p=0.087) and visuoconstructional components (F=5.395, p=0.006, η2p=0.105). Post hoc analysis revealed lower memory scores in MCI than SCD participants and in visuospatial abilities between MCI and SCD and MCI and Normal participants.

**Conclusions:**

We observed differentiated cognitive profiles among the participants in memory and visuoconstruction components. The use of PCA in the neuropsychological evaluation could help to make a differentiation of cognitive abilities in preclinical stages of dementia.

## RESUMO.

Os estágios pré-clínicos da demência incluem mudanças neurocognitivas sutis que não são facilmente detectadas nas avaliações clínicas padrão. A avaliação neuropsicológica é importante para a classificação e predição da deterioração em todas as fases da demência.

**Objetivo:**

Comparar o desempenho neuropsicológico em idosos saudáveis com declínio cognitivo subjetivo (DCS) e com comprometimento cognitivo leve (CCL) por meio da análise de componentes principais.

**Métodos:**

Avaliaram-se 94 idosos com um protocolo clínico que incluía medidas gerais do estado mental, emocional e funcional. O protocolo neuropsicológico incluiu tarefas de memória, função executiva, atenção, fluência verbal e habilidades visuoconstrutivas. Utilizou-se a análise de componentes principais (PCA, na sigla em inglês) para reduzir a dimensionalidade das variáveis na avaliação neuropsicológica.

**Resultados:**

Um total de 33 (35%) participantes apresentavam função cognitiva normal, 35 (37%) declínio cognitivo subjetivo e 26 (28%) comprometimento cognitivo leve. A PCA apresentou sete fatores: velocidade de processamento, memória, visuoconstrução, fluência verbal e componentes executivos de flexibilidade cognitiva, controle inibitório e memória de trabalho. ANOVA mostrou diferenças significativas entre os grupos na memória (F=4,383, p=0,016, η2p=0,087) e componentes visuoconstrutivos (F=5,395, p=0,006, η2p=0,105). A análise *post hoc* revelou escores de memória mais baixos no CCL do que os participantes com DCS e nas habilidades visuoespaciais entre CCL e DCS e CCL e participantes normais.

**Conclusões:**

Observaram-se perfis cognitivos diferenciados entre os participantes nos componentes de memória e visuoconstrução. O uso da PCA na avaliação neuropsicológica poderia auxiliar na diferenciação das habilidades cognitivas em estágios pré-clínicos da demência.

## INTRODUCTION

There is a progressive aging of the population in low- and middle-income countries. It is expected that, by 2050, 80% of aged people are expected to live in these countries.^1^ As a consequence, health care challenges are faced especially in the detection, follow-up, and treatment of neurodegenerative diseases, particularly those associated with dementia, which not only affect the patient’s quality of life but also their families and the entire social and health system.^2^

In Peru, there is a 6.85% prevalence of dementia, being more frequent in those who are illiterate (15%), and 60 to 70% are cases of Alzheimer disease (AD).^3^ AD biomarkers develop 15 to 20 years earlier than clinical symptoms^4^ and go through several stages that include subjective cognitive decline (SCD) and mild cognitive impairment (MCI). Recent studies in Peru, based on brief cognitive tests, have estimated that 17.9% of older adults have amnesic mild cognitive impairment (aMCI),^5^ but there are no data on the prevalence of SCD.

During the SCD phase, performance in cognitive tests is within the expected for the normative age group. However, there is a considerable concern about their cognitive condition regarding their previous normal status.^6^ It has been reported that approximately 25% of healthy adults with SCD will develop MCI^7^ with decline in episodic memory, executive function,^8^ and visuospatial functions;^9^ while the linguistic and attention skills will be preserved.^10^ On the other hand, MCI is considered a prodromal phase of dementia,^11^ which is characterized by alterations in more than one cognitive function (<1.5‒2 standard deviations related to the norm group) without having functional alterations.^12^ The percentages of progression to dementia range from 3 to 36%;^13^ however, people diagnosed with aMCI are more likely to develop dementia.^14^

To detect cases in different stages of AD, cerebrospinal fluid (CSF) biomarkers (tau protein and beta-amyloid) and neuroimaging (by magnetic resonance imaging - MRI and positron emission tomography - PET) have been used. However, these techniques are invasive, expensive, and not easily accessible.^15^ Currently, neuropsychological evaluation is the most used measure in clinical and research contexts for the detection of cognitive changes in the preclinical stages of dementia.^16^ ^,^ ^17^ ^,^ ^18^ It has been proven to have high sensitivity for the detection of subtle changes.^19^

These changes could be better detected by applying methods that reduce the number of factors of neuropsychological measures, especially principal component analysis (PCA).^20^ These dimensionality reduction techniques remove uninformative and redundant variables,^21^ extracting factors based on measurements and outcomes with tests in all cognitive domains. The factors extracted could help to classify the progression of deterioration into preclinical stages of dementia more accurately than individual tests, which could show considerable variation in their scores due to their complexity and difficulty of comprehension, mostly in contexts where literacy level is low, and lack of adapted scales and few specialized health practitioners and clinical centers.^2^ ^,^ ^22^

Therefore, these composite factors have been most sensitive to subtle cognitive changes in cognition that are not easily detectable independently.^23^ Furthermore, these composed factors are more sensitive in preclinical stages than the classic clinical tests such as the Mini-Mental State Examination (MMSE), the Alzheimer’s Disease Assessment Scale-Cognitive Subscale (ADAS-Cog) or the Clinical Dementia Rating (CDR). This is because the latter are not sensitive to mild changes related to episodic memory and executive function,^24^ which may have been affected in the first stages of the decline.^25^ Likewise, it has been shown that composite scores are good predictors in follow-up studies^26^ and clinical trials,^27^ and ease the communication in the clinical context.^28^ Recent studies have associated these composite factors to biomarkers of tau protein and beta-amyloid in different stages of the neurodegenerative process.^26^

From this perspective, neuropsychological evaluation is relevant to establish cognitive characteristics, especially in the preclinical stages of dementia, and to determine a neuropsychological profile, sensitive to the decline of aged adults. Consequently, this research aims to compare the neuropsychological performance of participants in preclinical stages of dementia using principal components analysis.

## METHODS

### Participants

The sample consisted of 94 adults from senior citizen clubs in the city of Arequipa, Peru, with ages ranging from 55 to 84 years old (M=65; SD=6.95). The participants were selected according to the following criteria: no history of alcohol and drug abuse; no previous psychiatric or neurological disease; and no severe perceptive deficits. Additionally, to include participants in the group of SCD, a question was asked about the current cognitive state regarding the previous status. In the case of the MCI group, participants with more than 7 years of education should score <27 in the MMSE, those with 4 to 7 years of education should have <23 points, and those participants with 1 to 3 years of education should have <21 score.^29^ Participants that did not have mood or functional alteration were also included (Table 1).

**Table 1.** Sociodemographic and clinical data of preclinical groups (n=94).

	Pre-clinical groups			p-value
	NORMAL n=33	SCD n=35	MCI n=26	
Male, n (%)	6 (40%)	3 (20%)	6 (40%)	
Female, n (%)	27 (34.2%)	32 (40.5%)	20 (25.3%)	
Age, mean (SD)	63.03 (5.45)	66.74 (7.13)	65.54 (7.09)	0.067+
Years of schooling, mean (SD)	12.92 (2.63)	13.31 (2.88)	12.62 (2.54)	0.651+
MMSE, mean (SD)	29.24 (0.83)	29.09 (0.74)	25.54 (1.83)	0.00**+
Diabetes, n (%)	4 (12)	2 (5.7)	5 (19.2)	0.266++
Cardiovascular disease n (%)	5 (15.2)	13 (37.1)	8 (30.8)	0.118++
Daily Exercise, n (%)	10 (30.3)	5 (14.7)	4 (15.4)	0.438++
Exercise 2-3 week, n (%)	12 (36.4)	12 (35.3)	11 (42.3)	
Never Exercise, n (%)	11 (33.3)	17 (50.0)	11 (42.3)	

MMSE: Mini-Mental State Examination; SCD: subjective cognitive decline; MCI: mild cognitive impairment; SD: standard deviation; **p<.001; ++Chi-square; +ANOVA.

### Procedures

The evaluations were made in 2 phases. In the first phase, the clinical protocol was applied to all the participants through the Peruvian version of the MMSE,^29^ the Beck Depression Inventory,^30^ the Yesavage geriatric depression scale,^31^ the Functional Activities Questionnaire (PFAQ),^32^ the Pfeiffer functional scale,^33^ and the sociodemographic and clinical health information, excluding those participants who did not met the inclusion criteria.

In the second phase, a neuropsychological protocol was used according to the guidelines of Ibáñez et al.^34^ and those that were standardized and validated in Perú.^35^ These protocol include episodic memory tasks with the Free and Cued Selective Reminding test (FCSRT), the Hopkins verbal learning test; attention and executive function tasks with the Symbol Digit Modalities Test (SDMT), modified version of Wisconsin Card sorting test (m-WCST), Trail making test A and B (TMT A-B), Stroop test and forward and backforward digits of the Weschler intelligence scale;^36^ verbal fluency with the phonological and semantic fluency tasks and visuospatial abilities with the Rey Osterich complex figure (ROCF). Additionally, we used the CDR to evaluate the clinical condition of the participants.^37^

Finally, the diagnoses of MCI were made based on the criteria of Albert et al.^12^ by neurologists and neuropsychologists. For the case of SCD, the criteria of the working group of the subjective cognitive decline initiative of the Alzheimer’s Association were taken into consideration:^38^ persistent experience of decline in the cognitive capacity in comparison to the previous state and not related to the recent events and regular performance on standardized cognitive tests that are used to classify the MCI.

### Statistical analysis

PCA was conducted to reduce the neuropsychological factors. Direct scores were transformed into Z scores for standardized data. Participants who obtained more than +/- 3 Z scores were retired for posterior analysis. A bivariate correlation was used to test the linear relationship between variables, and only significant associations were considered for analysis. Before PCA procedure, the Kaise-Meyer-Olkin (KMO) measure of sampling adequacy and the Bartlett’s test of sphericity were performed. Likewise, Varimax rotation for PCA analysis was used and items with factor loadings >0.4 were entered into a factor. For the number of principal components (PC) extracted the standard eigenvalues >1 criterion was considered.

With the components taken, the scores were compared according to the cognitive status (normal, SCD, and MCI) based on the analysis of variance (ANOVA). In the cases where significant differences were found, a post hoc analysis was also carried out with Bonferroni correction. Finally, a covariance analysis (ANCOVA) was conducted to evaluate the effect of years of education and age as covariates on those components that were significant.

### Ethical aspects

All participants were informed about the study and the criteria used in the study were those of the declaration of Helsinki for studies with human beings. The data were codified and recorded in such a way that only the principal researcher had access to the study and the other researchers gave blinded diagnostics.

## RESULTS

The participants’ characteristics were observed depending on their cognitive status (Table 1). No significant differences were found in the sociodemographic and clinical characteristics of the participants except for the scores of the MMSE, where patients with MCI had lower scores.

### Principal components extracted

The KMO index was 0.732 and Bartlett’s sphericity (χ^2^=1,732.47, p<0.000) indicated that data were suitable for factor analysis. The principal component analysis showed a solution of 7 factors: processing speed, memory, visuoconstruction, verbal fluency and the executive components of cognitive flexibility, inhibitory control, and working memory (Table 2). There were factorial weights ranging from 0.461 to 0.917 and an explained variance of 73%. SDMT and TMT A tests didn’t reach the factor loadings and were retired of the final model.

**Table 2.** Components loadings in extracted factors.

Neuropsy-chological test \ Components	Processing Speed	Memory	Verbal Fluency	EF Cognitive Flexibility	EF Inhibitory control	EF workingmemory	Viso construction
Stroop word	0.820						
Stroop Color	0.852						
Stroop W-C	0.917						
TMT B time	-0.533						
Free recall 1		0.774					
Free recall 2		0.839					
Free recall 3		0.826					
Delayed recall		0.779					
Fluency F			0.752				
Fluency A			0.799				
Fluency S			0.816				
Fluency Animals			0.676				
Fluency Fruits			0.461				
M-WCST NumTCat				-0.899			
M-WCST pe				0.777			
M-WCST NONpe				0.796			
TMT B errors				0.699			
Stroop W-C					0.829		
Stroop interference					0.955		
Digits forward						0.884	
Digits backward						0.649	
Rey Complex Figure Copy							0.793
Rey Complex Figure Memory-1							0.764

EF: executive function; M-WCST: Modified Wisconsin card sorting test; NumTCat: total number of categories; pe: perseverative errors; NONpe: nonperceverative errors; TMT B: Trail Making Test B; Stroop WC: stroop word color.

### Differences in clinical samples based on the cognitive state

The ANOVA applied to the collected factors showed significant differences in the PC of memory (F=4.383, p=0.016, η^2^p=0.087) and visuoconstruction (F=5.395, p=0.006, η^2^p=0.105), based on the cognitive condition (Table 3), in both cases the scores of the group of participants with MCI were lower than others. A post hoc analysis showed significant differences between participants with SCD and MCI in the memory component (t=2.919, p=0.012) and between participants with normal cognition and MCI (t=3.230, p=0.005), and SCD and MCI (t=2.406, p=0.047) of the visuoconstructional component.

**Table 3.** ANOVA for comparisons between groups according to components extracted.

	Total	Pre-clinical groups			p value	Post hoc comparisons
		NORMAL n=33	SCD n=35	MCI n=26		
		M (SD)	M (SD)	M (SD)		
Processing speed	0.02 (1.00)	0.26 (1.00)	-0.19 (1.06)	0.01 (.89)	0.117	N.A.
Memory	-0.00 (1.02)	0.06 (1.04)	0.27 (.84)	-0.48 (1.08)	0.016*	SCD>MCI
EF, cognitive flexibility	-0.02 (.98)	-0.01 (1.02)	-0.10 (1.01)	0.09 (.89)	0.758	N.A.
EF, inhibitory control	-0.03 (.97)	-0.30 (.87)	0.05 (.78)	0.20 (1.26)	0.114	N.A.
EF, working memory	-0.03 (1.00)	-0.04 (1.09)	0.05 (.99)	-0.13 (.89)	0.779	N.A.
Visoconstruction	-0.03 (1.00)	0.28 (.70)	0.00 (1.04)	-0.52 (1.13)	0.006*	NOR>MCI SCD>MCI
Verbal fluency	-0.02 (.98)	0.13 (.99)	-0.19 (1.02)	0.02 (.90)	0.392	N.A.

EF: executive function; SCD: subjective cognitive decline; MCI: mild cognitive impairment; NOR: normal; M(SD): mean (standard deviation); *p<0.05.

### ANCOVA of sociodemographic variables in memory and visuoconstruction factors

**Figure 1.**
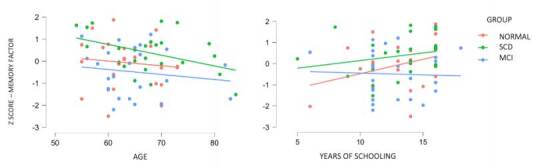
Descriptive plots of memory factor in pre-clinical groups by age and years of schooling.

Abbreviations: SCD: subjective cognitive decline; MCI: mild cognitive impairment.

It is observed that age and years of education do not have any significant effect on the memory component (F=1.223, p=0.273; F=1.535, p=0.220, respectively) or on the visuoconstructional component (F=2.974, p=0.089; F=1.371, p=0.246, respectively). On the other hand, as expected, the cognitive status (normal, SCD or MCI) shows a significant effect on the scores in the memory component (F=5.281, p=0.007, η^2^p=0.128), where aged participants, regardless of their cognitive status, seem to show a tendency to having a lower score, while normal participants and SCD show higher scores associated to the years of education. Participants with MCI appear to have lower and slightly decreasing scores to more years of education (Figure 1).

**Figure 2.**
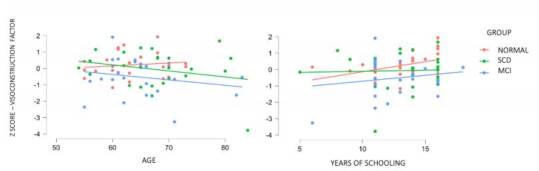
Descriptive plots of visoconstructional factor in pre-clinical groups by age and years of schooling.

Abbreviations: SCD: subjective cognitive decline; MCI: mild cognitive impairment.

Finally, it is observed that the cognitive status (normal, SCD or MCI) has a significant effect on the visuoconstructional component (F=3.528, p=0.035, η^2^p=0.089). Aged participants seem to have a better performance in visuoconstruction, though that does not happen with participants with cognitive decline and MCI (Figure 2). In addition, the years of education seem to be associated with better performance in visuoconstructional component, regardless of the cognitive status (Figure 2).

## DISCUSSION

The objective of this research was to compare the neuropsychological characteristics in preclinical stages of dementia based on principal component analysis. The results showed significant differences in the PC of memory and visuoconstruction in aged adults with normal cognition, SCD and MCI. Post hoc analysis showed lower memory scores in participants with MCI in comparison to participants with SCD, and no difference was found between the latter and participants with normal cognition. Finally, these differences in memory and visuoconstructional components remained unchanged after controlling age and years of education factors.

These results allow distinguishing the participants with normal cognition and SCD from the participants with MCI in memory and visuoconstructional processes, as demonstrated by other studies.^25^ ^,^ ^39^ But they also allow to see the tendency in the progressive decline during these phases seemingly unaffected by age and years of education. However, participants with MCI showed a tendency to have lower scores after more years of education. These results are consistent with other studies where changes in memory and visuospatial skills have been corroborated with the presence of CSF biomarkers^40^ or in patients with genetic mutation,^39^ showing greater deposits of tau protein and beta-amyloid in the entorhinal areas, connected with hippocampal and parietal medium structures.^41^ Additionally, some studies^17^ ^,^ ^42^ revealed a decrease in measurements of episodic memory and visuoconstruction in participants with SCD. Other authors have found decreases in more general visuospatial skills,^9^ which is why even these visuospatial skills have been considered to be better predictors of preclinical Alzheimer.^43^

A longitudinal study by Papp et al.^23^ using PCA to asses subtle decline in the cognitive tests in asymptomatic subjects reported variations (between -0.14 and -0.026 standard deviation per year) in subjects with positive beta-amyloid. Therefore, there is an up to 5 times greater risk associated to cognitive abilities changes, that could progress between 3 to 7 years prior MCI diagnosis and from 1 to 11 years prior to dementia diagnosis, mostly in verbal memory, visuospatial ability, executive function, and fluency.^42^ This data shows the importance of carrying out cognitive evaluations for an early diagnosis and follow-up of cases, especially in the subtle changes that are not easily detectable in routine clinical evaluations or population studies.^7^ ^,^ ^16^

This study has some important limitations. Firstly, since it was a cross-sectional study and based on clinical judgment for the diagnosis, there is a possibility of having wrong classifications in the study groups (controls, SCD, and MCI). Especially considering that, there is no evidence of biomarkers or neuroimaging that confirm the diagnosis or the presence of pathology. However, the diagnoses were based on a comprehensive evaluation and in agreement with a multi-disciplinary equipment of experimented clinicians where the structured clinical diagnosis served as a gold standard for this study. Furthermore, clinicians were blinded to the results of the first phase in order to prevent biases and over-estimation of diagnosis’ accuracy. It is also important to consider the context of the assessments. That is, part of the participants were evaluated at the beginning of the pandemic of Sars-Cov-19 and may have been affected on an emotional level;^44^ thus, performing a follow-up on them would provide a more accurate assessment and control for possible changes in the scores. Equally, nearly 85% of the sample was composed by women, which may have influenced the results.^45^ ^,^ ^46^.

Finally, we used a limited number of tests and outcomes for each domain. It is necessary to increment the numbers of tests to capture all the subdomains of cognitive processes and to improve differentiation in pre-clinical phases of dementia. In many cases, a single subdomain or test does not reflect all the cognitive process and could result in errors in patient’s classification. A composite factor can help reduce those errors, grouping all subdomains and outcomes in one factor. These facilitate the communication in clinical settings where health practitioners are not well trained on cognitive models or do not have standardized tests, like in some Latin American countries.^2^

This study showed that PCA is a valuable tool for the cognitive classification of deterioration in preclinical stages of dementia and the findings on the progression of changes in memory and visuospatial processing. Therefore, the decrease of cognitive dimensions through PCA has shown to be sensitive for the detection of changes.^47^ These findings may be associated to the progression of the pathophysiological alterations through the deposit of tau protein and beta-amyloid in medial-temporal and medial-parietal regions,^41^ especially in the precuneus, as demonstrated in recent works on visuospatial working memory.^48^ Therefore, it is considered that PCA may be a valuable tool to achieve the harmonization of the diagnostic criteria in the preclinical stages of dementia and in longitudinal studies.^6^ ^,^ ^8^ ^,^ ^38^

In conclusion, we observed differentiated cognitive profiles among the preclinical phases of dementia in memory and visuoconstructional abilities. PCA is very useful for classification and differentiation in preclinical stages and for the harmonization of criteria, the follow-up and classification of patients and the research of neuropsychological profiles sensitive to subtle changes in cognition. We strongly recommend delving into the cognitive profiles derived from PCA that may suggest the clinical utility of the evaluation in the memory and visuospatial domains, which could be added to studies with confirmatory biomarkers of neurodegenerative pathology.
